# The BMP Ligand Gdf6 Prevents Differentiation of Coronal Suture Mesenchyme in Early Cranial Development

**DOI:** 10.1371/journal.pone.0036789

**Published:** 2012-05-31

**Authors:** Dawn E. Clendenning, Douglas P. Mortlock

**Affiliations:** Department of Molecular Physiology and Biophysics, Center for Human Genetics Research, Vanderbilt University School of Medicine, Nashville, Tennessee, United States of America; Academia Sinica, Taiwan

## Abstract

*Growth Differentiation Factor-6* (*Gdf6*) is a member of the Bone Morphogenetic Protein (BMP) family of secreted signaling molecules. Previous studies have shown that *Gdf6* plays a role in formation of a diverse subset of skeletal joints. In mice, loss of *Gdf6* results in fusion of the coronal suture, the intramembranous joint that separates the frontal and parietal bones. Although the role of GDFs in the development of cartilaginous limb joints has been studied, limb joints are developmentally quite distinct from cranial sutures and how *Gdf6* controls suture formation has remained unclear. In this study we show that coronal suture fusion in the *Gdf6−/−* mouse is due to accelerated differentiation of suture mesenchyme, prior to the onset of calvarial ossification. *Gdf6* is expressed in the mouse frontal bone primordia from embryonic day (E) 10.5 through 12.5. In the *Gdf6−/−* embryo, the coronal suture fuses prematurely and concurrently with the initiation of osteogenesis in the cranial bones. Alkaline phosphatase (ALP) activity and *Runx2* expression assays both showed that the suture width is reduced in *Gdf6+/−* embryos and is completely absent in *Gdf6−/−* embryos by E12.5. ALP activity is also increased in the suture mesenchyme of *Gdf6+/−* embryos compared to wild-type. This suggests *Gdf6* delays differentiation of the mesenchyme occupying the suture, prior to the onset of ossification. Therefore, although BMPs are known to promote bone formation, *Gdf6* plays an inhibitory role to prevent the osteogenic differentiation of the coronal suture mesenchyme.

## Introduction

The mammalian cranial vault is composed of five main flat bones separated by joints known as the cranial sutures. These sutures are composed of fibrous connective tissue and act as the main sites for cranial growth during development. As the cranial vault expands, bone is deposited at the growing edges of the bone (the bone fronts), while the suture mesenchyme remains undifferentiated. Sutures provide flexible joints for passage through the birth canal, act as shock absorbers, prevent separation of the cranial bones, and accommodate room for the rapidly growing brain [1]. With the exception of the metopic suture, human sutures normally do not fuse until the third or fourth decade of life [2], when the undifferentiated mesenchyme of the suture space becomes obliterated by bone.

Craniosynostosis is defined as the premature fusion of one or more of the cranial sutures and occurs in approximately 1 in 2,500 live births [3]. When a suture fuses prematurely, cranial growth ceases perpendicular to the fused suture, producing a dysmorphic skull shape. In turn, when the calvarial vault cannot expand sufficiently to accommodate the rapidly growing brain, increased intracranial pressure can occur [4]. Coronal craniosynostosis can result from several potential mechanisms. For example, a failure to form the developmental boundary between the neural crest-derived frontal bone and the paraxial mesoderm-derived parietal bone can result in impaired suture formation. This failed mechanism is evident as a mixing of the frontal and parietal cell populations at sites of suture fusion in utero, as seen in the *Twist1* mutant mouse [5]. It is thought that *Twist1* functions with *Msx2* to control the localization of ephrin-A2 and ephrin-A4, which are known to play roles in boundary formation at the frontal/parietal junction by restricting cell migration [5]. Several additional mechanisms could also lead to fusion of a cranial suture. These include changes in proliferation, apoptosis, or the rate of differentiation in the suture mesenchyme or at the leading edges of the ossifying bone. For example, gain of function mutations in *Fibroblast growth factor receptors (FGFRs)* have been associated with craniosynostosis in humans. Studies in mice have shown that *Fgfr2* is expressed in proliferating osteoprogenitor cells surrounding the ossifying bones while *Fgfr1* is expressed more centrally in osteoid of the developing frontal and parietal bones. As differentiation progresses, *Fgfr2* is downregulated and *Fgfr1* is upregulated, suggesting that signaling through FGFR2 mainly plays a role in proliferation, while FGFR1 signaling regulates osteogenic differentiation. The contribution of FGFR3 is less clear though its expression overlaps with FGFR1 and −2 [6]. However, the P250R gain-of-function mutation in FGFR3 has been associated with coronal craniosynostosis, either isolated or as part of syndromes such as Muenke syndrome [7]. While defects in boundary formation between lineage compartments (e.g. neural crest and paraxial mesoderm) can explain some of the etiology of coronal craniosynostosis, it remains less clear how proposed changes in differentiation or maintenance can affect certain sutures while sparing others.

Growth Differentiation Factors (GDFs) 5, 6, and 7 are members of the Bone Morphogenetic Protein (BMP) family of secreted signaling molecules. The GDF subgroup (GDF5/6/7) is highly conserved in vertebrates and has been shown to play a critical role in limb joint formation and chondrogenesis [8]. *Gdf6* homozygous knockout mice display multiple joint defects, including fusions of tarsal and carpal bones, morphological abnormalities in the malleus, incus, and stapes bones of the middle ear, and hypoplasia of the thyroid cartilage. In addition to these defects, *Gdf6* knockout mice lack the coronal suture [9]. However, the detailed expression pattern of *Gdf6* in the developing skull and its relationship to the onset of cranial suture fusion in this mutant has not been shown. Another GDF family member, *Gdf5,* is mutated in the *brachypodism* mouse and normally stimulates cartilage development, growth, and maturation [8]. Therefore, *Gdf5* can be viewed as promoting aspects of endochondral bone growth. *Gdf5* and *Gdf6* share approximately 80% identity in the mature signaling region [10] and therefore it is likely the *Gdf5* and *Gdf6* operate by similar ligand-receptor interactions. Like *Gdf5, Gdf6* (a.k.a. CDMP2) can promote chondrogenic differentiation in vitro [11]. This makes the craniosynostosis phenotype in the *Gdf6*−/− mouse particularly interesting because unlike the long bones, the cranial bones form through intramembranous ossification without a cartilage intermediate. Therefore, the mechanism of *Gdf6* function in the coronal suture may be drastically different than its function in joints of the axial skeleton.

The aim of this study was to gain a more thorough developmental understanding of craniosynostosis in the *Gdf6−/−* mouse and the underlying cause of suture fusion. We found that the coronal suture is obliterated in *Gdf6−/−* embryos before the first evidence of cranial bone ossification is detectable at E14.5, with changes in early osteogenic markers detected prior to the onset of ossification. Our data suggests that *Gdf6* may self-regulate its expression in the developing frontal bone primordium. Additionally, fusion in the *Gdf6*−/− is not due to a failure to form the boundary properly between the frontal and parietal bones, or changes in cell survival or proliferation, but is likely due to a failure of the suture mesenchyme to remain in an undifferentiated state.

## Results

### *Gdf6−/−* Coronal Suture Fuses Early in Development

**Figure 1:**
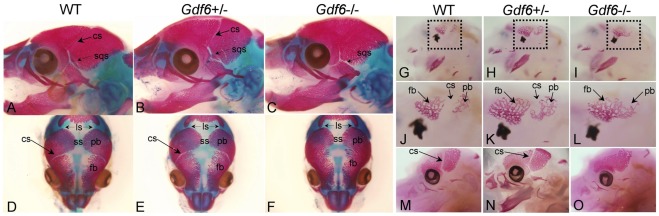
Analysis of coronal suture development in wild-type and *Gdf6−/−* embryos with alizarin red. The coronal suture separated the frontal and parietal bones in wild-type and heterozygote embryos (A, B), but was lacking in the *Gdf6*−/− embryos (C). At E14.5, the early coronal suture was a distinct gap between the frontal and parietal ossification centers (G, H, J, K). The coronal suture appeared fused in *Gdf6*−/− embryos at the onset of ossification (I, L). The frontal and parietal bones fused into one continuous bone in the *Gdf6*−/− at E15.5 (O). The sagittal, lambdoid (D–F), and squamosal (A–C) sutures remained unfused through weaning in both wild-type and *Gdf6*−/− animals. fb, frontal bone; pb, parietal bone, cs, coronal suture; ls, lambdoid suture; sqs, squamosal suture.

The entire coronal suture was absent in *Gdf6*−/− fetal mice (Fig. 1C, F), with complete penetrance (not shown). This defect was not observed in wild-type (Fig. 1A, D) and *Gdf6* heterozygote littermates (Fig. 1B, E) through embryonic development and weaning. To determine the time point during development at which the suture first became fused, *Gdf6−/−* embryos were collected at various stages and stained with alizarin red. At E14.0, ossification centers in either the frontal or parietal bone were not yet visible by alizarin red staining. By E14.5, frontal and parietal bones were first visible as two separate ossification centers (Fig. 1G, J; H, K). The nascent coronal suture was apparent in the wild-type embryo as the gap between the two bones. Yet in *Gdf6*−/− embryos, a single continuous ossification center was present (Fig. 1I, L). Analysis of multiple *Gdf6−/−* embryos at E13.5–14.5 failed to identify visibly separate sites of alizarin staining for frontal and parietal rudiments in any specimens (not shown). At E15.5, the frontal and parietal bones were fused into one continuous bone in the *Gdf6*−/− embryo (Fig. 1O), while the bones remained separated by the coronal suture in the wild-type and heterozygote (Fig. 1M, N) embryos. The coronal suture continued to fuse along its entire length as ossification progresses outward in the *Gdf6−/−* embryo, whereas the sagittal, lambdoid, and squamosal sutures (Fig. 1A–F) remained unaffected in *Gdf6*−/− mice throughout prenatal development. At the macroscopic level, the ossification centers and suture in *Gdf6+/−* appear to develop identically to wild-type embryos with regards to the onset of ossification and size of the bones (not shown). This data suggests that *Gdf6* plays a role in coronal suture formation at or prior to the onset of ossification.

### *Gdf6−/−* has Normal Suture Boundary Formation

Coronal craniosynostosis can result from a failure to form a proper boundary between cells of the neural crest-derived frontal bone and the paraxial mesoderm-derived parietal bone. The formation of this boundary involves the cooperation of *Twist1* and *Msx2* to control of the expression domains of ephrin-A2, ephrin-A4, and EphA4 [5]. Ephrin signaling has been shown to inhibit cell mixing and provide guidance cues for migrating cell populations [12]. Failed boundary formation is evident as a mixing of the two cell lineages within the suture mesenchyme, as in the *Twist1* mutant mouse [5].

**Figure 2:**
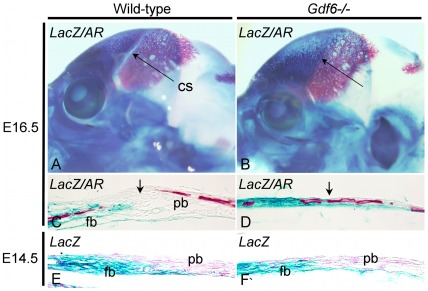
Analysis of boundary formation in *Gdf6*−/− embryos. E16.5 *Wnt1Cre+/−; R26R+/−; Gdf6+/+* (A) and *Wnt1Cre+/−; R26R+/−; Gdf6−/−* (B) embryos were stained with Xgal to label the frontal bone followed by alizarin red to highlight the parietal bone. Sections through the *lacZ*/alzarin red stained embryos show the boundary between the labeled frontal bone and unlabeled parietal bone at the site of the wild-type suture (C, arrow), with bone continuing through the suture boundary in the *Gdf6−/−* embryo (D, arrow). E14.5 *Wnt1Cre+/−; R26R+/−; Gdf6+/+* (E) and *Wnt1Cre+/−; R26R+/−; Gdf6−/−* (F) embryos were stained with Xgal and counterstained with nuclear fast red. In both the wild-type and *Gdf6−/−* coronal sutures, the boundary was distinct, with no evidence of cell mixing. AR, alizarin red; cs, coronal suture; fb, frontal bone; pb, parietal bone.

To determine whether a similar cell mixing was the cause of the craniosynostosis in the *Gdf6−/−* embryos, we visualized the suture boundary using the *Wnt1-Cre* and *R26R* transgenic lines, which together stably label derivates of the neural crest [13], including the frontal bone. At E16.5, coronal suture fusion was evident in whole-mount stained *Gdf6*−/− embryos (Fig. 2A,B). Transverse sections showed the presence of ossified bone across the boundary between the frontal and parietal bones in *Gdf6*−/− (Fig. 2D, arrow), while the suture remained open and undifferentiated in wild-type (Fig. 2C, arrow). Also of note was a general thinning of the bone in this region of the *Gdf6*−/− (Fig. 2D) along with the loss of the characteristic suture mesenchyme blastema, seen in wild-type embryos (Fig. 2C, arrow). In the regions of the frontal bone more distal to the suture, there was no thinning of the *Gdf6−/−* bone compared to wild-type (not shown).

In the *Gdf6−/−* embryo, ossified tissue disrupted the boundary between the frontal and parietal domains (as seen in Fig. 2D) making it difficult to determine if cells have crossed the normal boundary. Therefore embryos were examined at E14.5, when portions of the *Gdf6*−/− calvaria were not yet ossified through the suture. Coronal sections through the region of the presumptive coronal suture reveal that although the *Gdf6*−/− lacked an identifiable suture, the cellular boundary between the frontal and parietal bones remained distinct just like the wild-type suture (Fig. 2E,F). We could find no evidence of cell mixing between these tissue populations and there were no other obvious differences between wild-type and *Gdf6+/−* embryos with regards to the suture boundary (not shown). Furthermore, we observed a surprisingly uniform and continuous surface between the frontal and parietal bones of *Gdf6−/−*, where the suture should reside.

### Gdf6−/− *and* Gdf6+/− *Present with Pre-ossification Changes in the Suture*

**Figure 3:**
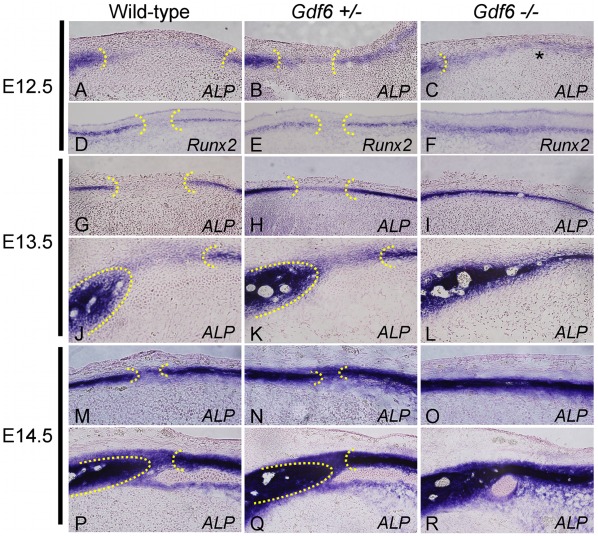
Alkaline-phosphatase (ALP) staining in the presumptive suture. Transverse sections through the E12.5 wild-type suture showed a gap in ALP activity between the frontal and parietal bones (A). The width of the gap was reduced in the *Gdf6*+/−, with an increase in ALP activity within the suture (B). In the *Gdf6*−/−, there is a continuous line of ALP activity through the region, with a reduction in ALP activity in the parietal portion of the fused bone (C). Similar changes in ALP staining were observed in the E13.5 and E14.5 rostral (G–I, M–O) and caudal (J–L, P–R) coronal sutures and through *in situ* hybridization for *Runx2* (D–F).

Transverse sections through the E12.5 wild-type suture were stained for alkaline phosphatase (ALP) activity, an early osteoblast marker, to highlight the frontal and parietal bone primordia with the presumptive coronal suture in between (Fig. 3A); ALP was weaker in the band of cells between the primordia, but was faintly visible across the suture region (Fig. 3A). In *Gdf6+/−* embryos, ALP activity extended through the presumptive suture region which was shorter as defined by the distance between the flanking bone primordia (Fig. 3B), revealing less undifferentiated suture mesenchyme relative to the wild-type suture. Although *Gdf6*+/− mice develop normal sutures, ALP was also increased in the presumptive suture relative to wild-type. This points to a dosage effect, where loss of one copy of *Gdf6* does increase ALP activity in the suture, but this slightly increased differentiation does not reach the threshold required for fusion of the suture. In addition, the suture gap width was reduced in the *Gdf6+/−* compared to wild-type (Fig. 3B). In E12.5 *Gdf6−/−* embryos, the frontal primordium was clearly visible, but there was no clear region of concentrated ALP in the parietal primordium; rather, there was continuous, moderate ALP staining across the region of the presumptive suture and extending into the region where the parietal primordia has concentrated ALP activity in wild-type and *Gdf6*+/− embryos (Fig. 3C).

Differentiation of the suture mesenchyme was also examined by expression of *Runx2*, another marker for early bone differentiation. Like ALP, *Runx2* marked the presumptive frontal and parietal bones, with a gap of undifferentiated mesenchyme in between (Fig. 3D). Again, the distance between the frontal and parietal rudiments was reduced in the *Gdf6+/−* (Fig. 3E). In the *Gdf6*−/−, *Runx2* was expressed continuously through the suture region at E12.5 (Fig. 3F). These data suggest the presumptive suture region of *Gdf6*−/− embryos is prematurely differentiating by E12.5, as characterized by abnormally high ALP and *Runx2* expression. Interestingly, ALP activity is apparently delayed in the parietal rudiment of *Gdf6*−/− embryos although *Runx2* expression is not.

Increased ALP activity was confirmed at both caudal (closer to the eye) and rostral (newly formed) levels of the presumptive coronal suture at E13.5 and E14.5 (caudal, Fig. 3J–L, P–R and rostral, Fig. 3G–I, M–O). This suggests an increase in osteogenic differentiation of the cells in the developing suture of the *Gdf6+/−* and *Gdf6−/−* embryos, and also a dosage effect where *Gdf6+/−* has intermediately increased differentiation between wild-type and *Gdf6−/−* embryos.

A decrease in both the intensity (Fig. 3C,I) and span (not shown) of ALP activity in the parietal bone rudiment of *Gdf6−/−* embryos was noted, suggesting a decrease in the rate of differentiation in this structure. This was also observed in the alizarin red staining of E14.5 embryos, with a reduction of the size of the *Gdf6−/−* parietal bone ossification center compared to wild-type and *Gdf6+/− (*not shown). This could be a potential secondary effect of loss of the suture, or a direct effect of the loss of *Gdf6*.

### Loss of Gdf6 does not Significantly Affect Proliferation in the Nascent Suture

**Figure 4:**
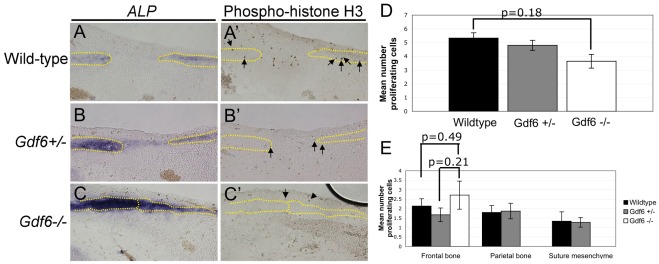
Analysis of cell proliferation in the E12.5 coronal suture. Adjacent sections stained for ALP activity, highlighting the location of the frontal and parietal bones (A–C dotted lines), and immunohistochemistry for phospho-histone H3 (A’–C’). Examples of positive cells are marked with arrows. (D) Quantification of the mean number of proliferating cells counted in the combined frontal/suture/parietal region. (E) Quantification of the mean number of proliferating cells counted in each region separately (the frontal bone, parietal bone, and suture mesenchyme) (x-axis). Since the suture mesenchyme cannot be distinguished from the parietal in Gdf6−/− embryos at this time, only data for the frontal rudiment is shown for this genoytpe. N = 3 embryos for each genotype with 5 sections per embryo quantified. Differences the number of proliferating cells per suture region were analyzed using a t-test.

In principle, suture fusion could result from changes in the number of cells in the suture or bone fronts proliferating or undergoing apoptosis, thereby increasing or decreasing the number of cells in the pre-osteogenic pool. Between E14.0 and E14.5, the frontal and parietal rudiments in the *Gdf6−/−* are clearly fused as shown by alizarin red staining into a single ossified element (Fig. 1I, L). Therefore the sutures of wild-type, *Gdf6+/−*, and *Gdf6−/−* littermates were examined for changes in proliferation or apoptosis at E12.5, concurrent with the earliest detectable changes in ALP staining (Fig. 3) but before the formation of osteoid. Adjacent sections were stained for ALP in order to help localize the suture (Fig. 4A–C). Immunohistochemistry for phospho–histone H3 (Fig. 4A’–C’) showed no significant overall changes in the number of proliferating cells in heterozygous or homozygous mutants as compared to wild-type, nor when the suture mesenchyme, frontal bone, and parietal bone were analyzed separately (Fig. 4D,E). Proliferation assays at E13.5 also failed to detect significant differences across genotypes (Fig. S1) although some trends toward altered proliferation rates in mutant mice were suggested; although not significant statistically, these trends may be secondary to the increased rate of differentiation in the mutant sutures and thus, to changes in the pool of pre-osteogenic proliferating cells.

To assay apoptosis, we used cleaved-caspase 3 immunohistochemistry. Few to no apoptotic cells were detectable in the suture mesenchyme or bone front in pre-ossified calvaria (Fig. S1), consistent with previous reports that there are very few apoptotic cells in fetal mouse calvaria prior to E16.5 [14] and likewise, no significant difference between genotypes was found.

### Gdf6 is Expressed in the Frontal Bone Primordia

**Figure 5:**
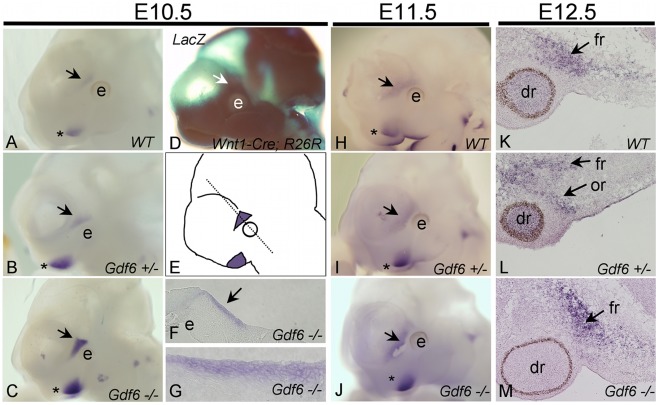
Expression of *Gdf6*. *In situ* hybridization for *Gdf6* at E10.5 (A–C, F,G), E11.5 (H–J), and E12.5 (K–M). *Gdf6* was expressed in the frontal bone primordia (arrow), which is labeled as neural crest-derived in the *Wnt1-Cre; R26R* transgenic embryos (D). *Gdf6* transcript was expressed more strongly in the *Gdf6−/−* embryo (C, J) than either the wild-type (A, H) or *Gdf6+/−* embryo (B, I), in both the frontal bone rudiment (arrow) and branchial arches (asterix). In transverse cross section through the eye and frontal bone primordia at E10.5 (dotted line, E), *Gdf6* was expressed in several layers of mesenchyme underlying the surface ectoderm (F, G). At E12.5, *Gdf6* continues to be expressed in the frontal bone rudiment, in addition to the dorsal retina (K–L) and orbital bone rudiment (L). Expression of *Gdf6* in the dorsal retina seen in the wild-type and *Gdf6+/−* (K,L) was absent in the *Gdf6−/−* (M). dr, dorsal retina; e, eye; fr, frontal rudiment; or, orbital bone rudiment.

The neural crest/paraxial mesoderm tissue boundary that determines the future site of the coronal suture is formed by E10.5 [13]. In order to pinpoint the pattern of *Gdf6* expression during cranial development and specifically in relation to coronal suture formation, we performed *in situ* hybridization on embryos at E9.5–E14.5. *Gdf6* mRNA was first detected in the developing cranial region in a triangular-shaped area just anterior to the eye at E10.5 (Fig. 5A–C, arrow). This corresponded closely with the neural crest-derived frontal bone rudiment, as labeled in *Wnt1-Cre; R26R* embryos (Fig. 5D, arrow). *Gdf6* was expressed in the frontal bone rudiment at E11.5 (Fig. 5H–J), as the rudiment begins to grow and expand. Transverse sections through the E10.5 frontal rudiment (depicted in Fig. 5E, dotted line) reveal that the site of expression anterior to the eye was localized to several layers of mesenchyme underlying the surface ectoderm (Fig. 5F,G). At E12.5, *Gdf6* continued to be exclusively expressed in the frontal bone rudiment (Fig. 5K–M, arrow), with no evidence of expression in the suture mesenchyme or parietal bone rudiment. By E14.5, when fusion of the ossification centers was first visible by alizarin red staining, *Gdf6* is no longer expressed in the suture region (Fig. S2).

Although no functional Gdf6 protein can be made from the *Gdf6* knockout allele, the mutant *Gdf6* transcript can be detected by our *in situ* RNA probe, which targets the 3′ UTR of *Gdf6*. *In situ* hybridization revealed that *Gdf6* transcripts were still present in *Gdf6*−/− embryos (Fig. 5C,J,M). Interestingly, the *Gdf6* transcript was more highly expressed in *Gdf6*−/− embryos than in either wild-type or *Gdf6+/−*, with staining in *Gdf6+/−* at intermediate levels (Fig. 5A–C, H–J). Increased staining was also observed for *Gdf6* in the branchial arches of *Gdf6−/−* embryos (Fig. 5, A–C, H–J asterix). These observations suggest that *Gdf6* expression may be self-regulated in these structures by a negative feedback loop. In cross-section, *Gdf6* expression was also observed in the orbital bone rudiment (Fig. 5L) of wild-type, *Gdf6+/−,* and *Gdf6−/−* embryos (not shown). *Gdf6* is also normally expressed in the dorsal retina where it functions in promoting eye development [15], [16]. However, *Gdf6* transcript was observed in the dorsal retina wild-type and *Gdf6+/−* embryos (Fig. 5K,L), but was lost in *Gdf6−/−* embryos (Fig. 5M), suggesting that in the retina *Gdf6* autoregulates through a positive feedback loop, in contrast to the negative autoregulation in frontal bone and branchial arches.

## Discussion

Here we present data indicating that *Gdf6* is genetically required for osteogenic differentiation in the developing coronal suture during its formation. *Gdf6* is absolutely required for formation of the suture, as in *Gdf6−/−* embryos there is an initial failure to establish a region of delayed differentiation between the frontal and parietal condensations. We observed that *Gdf6* mRNA is strongly downregulated in wild-type calvaria by E14.5. It remains unknown if *Gdf6* plays roles at later stages to maintain patency of the established suture; such a notion would be consistent with a report that *Gdf6* expression is detectable in calvarial sutures by E16.5 [9]. The question of whether *Gdf6* plays a role in suture maintenance will require examination of Gdf6 protein stability and localization, and/or conditional deletion of *Gdf6* at later stages.

### Gdf6 Represses Osteogenic Differentiation but not Boundary Formation in the Coronal Suture

We found no evidence for disruption in the frontal/parietal cell boundary in *Gdf6−/−* mice. This is in contrast to the mechanism of suture fusion in the *Twist+/−*, and *Epha4−/−* mice where, before E14.5, osteogenic cells from the frontal bone abnormally cross into the suture mesenchyme [5]. In *Twist+/−* mice, ALP activity in the frontal/parietal rudiments is normal in appearance up to at least day E13.5 [5]. In contrast, ALP activity abnormalities are detectable by E12.5 in *Gdf6−/−* embryos, before ephrin ligands are expressed in the frontal/parietal region [5]. Therefore *Gdf6* is required for a mechanism of suture formation that is distinct from that controlled by the *Twist/Ephrin* pathway. *Twist* also regulates osteogenic condensation via interaction with *Msx2* [17]. Our data does not exclude *Gdf6/Twist* interactions during osteogenic differentiation, although a combined reduction of *Twist* and *Msx2* levels was shown to primarily affect differentiation of the frontal, but not the parietal bone, suggesting this is possible [17].

### Effects of Gdf6 Signaling in the Developing Calvarium

Somewhat surprisingly, *Gdf6* mRNA was not detected in the suture mesenchyme itself but in the frontal bone primordia. This is in contrast to the sites of wrist and ankle joint fusion in *Gdf6−/−* embryos, where *Gdf6* is clearly expressed in the developing joint interzones [9]. However, several studies suggest that the action of GDFs in limb joint development is not explained by direct autocrine suppression of chondrogenesis; for example, both the direct application of GDF5 protein to developing limb cartilage and transgenic *Gdf5* overexpression are pro-chondrogenic [8], [18], although in limb joints the pro-chondrogenic effects of GDFs are probably inhibited by Noggin [19]. *Gdf5/6/7* form a closely related subfamily of BMPs, sharing >80% identity in their mature C-terminal signaling domains [10]. The strong similarity of *Gdf5* and *Gdf6* suggest they share similar signaling properties. Genetic evidence from analysis of *Gdf5/Gdf6* compound mutant mice supports the idea that they have similar, partly redundant roles in skeletal development that are determined largely by site of expression rather than distinct signaling mechanisms [20]. A unifying theme of both limb joints and the frontal bone is that both are important paracrine signaling centers for adjacent targets (that is, cartilage in the limb and the suture mesenchyme in the cranium). We propose that *Gdf6* expression in the frontal bone primordium enables it to serve as a paracrine signaling center to affect cellular processes in the suture mesenchyme and the parietal rudiment. It is not yet clear if this occurs via direct signaling or through indirect effects transmitted by downstream effectors.

Interestingly, the onset of ALP activity is delayed in the parietal, but not frontal, primordia in *Gdf6−/−* embryos. Since *Gdf6* is expressed in the frontal but not parietal primordia, this suggests *Gdf6* signaling from the frontal primordium also acts in a paracrine manner to influence maturation of the parietal. In this view, Gdf6 actually stimulates osteogenic maturation of the parietal rudiment, although it is not clear if this affect is direct or indirect (for example, it could be a secondary effect mediated by Gdf6 regulating a separate factor in the suture mesenchyme). Therefore, data regarding Gdf6’s possible roles in skeletal differentiation are important for interpretation of our results. Several reports indicate that like Gdf5, Gdf6 can stimulate chondrogenic differentiation in cell culture models [11]. Whether Gdf6 is pro- or anti-osteogenic is less clear. Some studies indicate that in vitro, Gdf6 can have pro-osteogenic effects on osteoblastic cells in similar manner to Gdf5, albeit its ability to induce osteoblast markers is much less than that of the “canonical” osteogenic BMPs such as BMP2 or BMP7 [21]. However, Gdf6 can inhibit ALP expression and mineralization in bone marrow-derived mesenchymal stem cells [22]. *In vitro* experiments must be interpreted with caution, due to potential differences in expression of BMP receptors and/or inhibitors across cell lines. In general, *in vitro* studies indicate that Gdf6 seems consistently capable of promoting differentiation of chondrogenic cells but relatively poor, or inhibitory, at promoting osteogenesis.

However, injection of GDF5 into perinatal mouse calvaria *in vivo* led to increased bone formation [23]. While this suggest GDFs can have stimulatory effects on calvarial osteogenic differentiation, the different timing and context of Gdf5 application may not lead to the same effects as Gdf6 in the prenatal calvaria and Gdf6 may have distinct signaling effects compared to Gdf5, despite similar receptor usage [24]. Other studies indicate that the effects of *Gdf6* on ALP induction are context dependent [25]. This effect might be mediated by receptor subunit combinations, interactions with inhibitors such as Noggin, or even heterodimerization with other BMP family members. Noggin is expressed in layers surrounding the developing frontal and parietal bones and can bind Gdf6 and inhibit its signaling ability [26]. Noggin can repress BMP signaling in the coronal suture [27], so it likely inhibits Gdf6-mediated signaling. However, Noggin is not required for suture formation, as *Noggin*−/− embryos do form coronal sutures (not shown). Therefore, Noggin is probably dispensable for the function of Gdf6 in suppressing suture differentiation. However, our data would not exclude the possibility that the reduction of ALP activity in the Gdf6−/− E12.5 parietal bone could be caused by increased Noggin activity, secondary to a reduction of Gdf6 that normally suppresses Noggin. Xenopus GDF6 can heterodimerize with other BMPs, such as BMP2, *in vivo* [26], [28] and other BMP heterodimers can have distinct and potent effects as compared to homodimers [29]. Therefore it is possible that Gdf6/BMP heterodimers have unique signaling properties.

Alternatively, it is possible that the relative temporal development of the frontal and parietal rudiments is critical, and that the relative delay of the parietal ossification sequence leads indirectly to failure of suture establishment. However, the onset of *Runx2* transcription is not delayed in *Gdf6−/−* embryos, suggesting *Gdf6* is not required for temporal control of *Runx2* mRNA in calvarial rudiments. We postulate that the suture mesenchyme and the parietal rudiment may be differentially sensitive to *Gdf6* signaling.

### Gdf6 Autoregulation and Interaction with other BMPs

We observed that *Gdf6* transcription is increased in frontal bone, but reduced in eyes, of *Gdf6−/−* embryos. This suggests differential, tissue-specific autoregulation of *Gdf6*. Interestingly, *Gdf5* represses its own transcription in limb joints [8]. We propose that *Gdf6* autoregulates itself in the frontal bone and that Gdf6/BMP signaling levels are fine-tuned during normal coronal suture development to coordinate proper differentiation and morphogenesis. Interestingly, *Bmp4* and *Gdf6* are coexpressed in both the dorsal retina and the frontal bone primordia [16]. Mutations in *Bmp4* and *Gdf6* independently disrupt eye development [15], [30], [31]. Although *Gdf6* is expressed in the frontal bone rudiment, there is no evidence for a frontal bone defect in *Gdf6−/−*. This is likely due to compensation by *Bmp4*. *Bmp4+/−; Gdf6−/−* mice at E18.5 do in fact have a frontal bone defect, with the persistence of large fontanelles that is not observed in either single heterozygotes (data not shown; manuscript in preparation). We speculate that these two BMP ligands cooperate to regulate suture and/or calvarial development.

### Conclusions

In summary, we found that *Gdf6* is required to control an early stage of repressed osteogenic differentiation in the coronal suture. Not only does this suggest potential new mechanisms for this BMP family member in regulating bone growth, it nominates *GDF6* as a candidate gene harboring mutations in individuals with coronal craniosynostosis. *GDF6* mutations in humans have been associated with eye and postcranial skeletal abnormalities although these effects are characterized by incomplete penetrance and phenotypic heterogeneity [15], [32]. Interestingly, genomic lesions close to the *GDF6* genomic region are associated with Nablus Mask-Like Facial Syndrome [33], [34], [35] which is a complex multigene deletion syndrome characterized by loss of a critical region just proximal to *GDF6*. In one of only two known patients where the genomic deletion included *GDF6*, coronal craniosynostosis was observed [33], [34]. We propose that some individuals having coronal craniosynostosis with unknown etiology may harbor mutations in *GDF6*.

## Materials and Methods

### Mice, Crosses and Genotyping

The *Gdf6*−/− mouse [9] was a gift from Dr. David Kingsley and was backcrossed onto a C57BL/6J background for more than 10 generations. Since the loss of *Gdf6* is perinatal lethal on this background (not shown), all time points were collected prenatally. For fate mapping experiments, *Gdf6+/−* mice were crossed to *R26R*+/− [36] to produce *Gdf6+/−; R26R+/−* (double heterozygotes), which were then crossed to *Wnt1Cre*+/− [37]; *Gdf6*+/− mice. Embryonic age was determined through detection of the vaginal plug, with noon of that day observed as E0.5. DNA samples for genotyping were isolated using tail snips from postnatal mice and yolk sacs from embryos, and processed as previously described [38]. The *Gdf6* [9], *R26R* [36], and *Wnt1-Cre* [39] lines were genotyped by PCR analysis as previously described. The use of animals in this study was approved by the Vanderbilt University Institutional Animal Care and Use Committee as part of animal use protocol M/09/293, approved on 1/25/10 and 1/25/11.

### Whole Mount Skeletal Preps

Mid-gestation mice from E14.5 to E18.5 were collected, organs removed and skinned. Each specimen was placed in a separate 50 ml conical tube and then soaked in 95% ethanol for 1 day. Specimens were agitated constantly on a shaker. Preps were then stained in alcian blue solution [20% glacial acetic acid, 0.015% alcian blue in 95% ethanol] for 14 days, destained in 95% ethanol for 1 day, then transferred into 1% KOH until cleared. Once cleared, the skeletal preps were placed in alizarin red staining solution [0.00125% alizarin red in 1% KOH] for 1 day. Skeletal preps were then transferred into graded changes of increasing glycerol for storage (15%, 30%, 50%, 70%, 90% glycerol made in 1× PBS, 100% glycerol).

### Whole Mount and Slide *in situ* Hybridization

The *Gdf6* RNA probe was generated by cloning a PCR fragment using the primers 5′- AAGCATGGAAGGAGGATGAAAGGG- 3′ and 5′- CGACCTCCAGTAACTTTAGTGTTGTCA –3′, targeting the *Gdf6* 3′ untranslated region, into the pGEM-Teasy vector (Promega). The *Runx2* RNA probe was made using the primers and protocol described previously [40]. Hybridization was performed over night at 63°C, with embryos incubated in 200 ng/ml (whole-mount) or 30 ng of probe per slide (sections). For sectioning, embryos were equilibrated in 50% sucrose and then embedded in Tissue-Tek O.C.T. compound (Sakura Finetek). Frozen sections were collected at 18 um. For each time-point, embryos were from the same litter and stained for an equal length of time.

### Histology

Embryos were dissected in 1× PBS and fixed for 60 min at 4° in 10% neutral buffer formalin (Sigma), decapitated, bisected sagittally, the skin removed, and fixed for another 15 min. Embryos were prepared for X-Gal staining as previously described [38]. Whole-mount specimens were further stained with alizarin red overnight. Sectioned specimens were dehydrated through ethanol series and embedded in paraffin. 10 µM sections were collected and counterstained with nuclear fast red (Vector Laboratories).

For staining for ALP activity, frozen sections were collected at 18 um and stored at –80°C until ready for staining. Slides were brought to room temperature, then washed in acetone, TBST [Tris-buffered saline pH 8.0, 1% Tween-20], and NTMT [0.1 M NaCl, 0.1 M Tris-HCl pH 9.5, 50 mM MgCl2, 0.1% Tween-20] at 4°C, stained with nitro-blue tetrazolium chloride (NBT) and bromo-4-chloro-3′-indolyphosphate p-toluidine (BCIP) at room temperature, then counterstained with nuclear fast red.

Immunohistochemistry for Phospho-Histone-H3 (Ser10) and Cleaved Caspase-3 (Asp175) was carried out on cryosections. Slides were fixed in neutral buffer formalin, treated with 0.3% H_2_O_2_ to quench endogenous peroxidase activity, and blocked with 5% normal goat serum in PBS. Diluted primary antibody (Phospho-Histone-H3 1∶200, Cleaved Caspase-3 1∶12,800) was applied overnight at 4°C. Sections were developed using a biotinylated rabbit secondary antibody with ABC solution (Vector Laboratories) and DAB.

## Supporting Information

Figure S1 **Analysis of cell proliferation and apoptosis in the coronal suture.** Adjacent sections stained for ALP activity, highlighting the location of the frontal and parietal bones (A–F dotted lines), and antibodies for phospho-histone H3 (A’–C’) or cleaved caspase-3 (D’–F’). Positive cells are marked with arrows. (G) Quantification of the mean number of proliferating cells (y-axis) counted in the suture region (x-axis). (H) Quantification of the mean number of proliferating cells (y-axis) counted in each region of the suture; the frontal bone, parietal bone, and suture mesenchyme (x-axis). The mean number of proliferating cells in each region of the suture was not counted for *Gdf6−/−* embryos since there is no suture mesenchyme and the border between the frontal and parietal bones cannot be distinguished. N = 3 embryos for each genotype and antibody treatment with 5 sections per embryo quantified. Differences the number of proliferating cells per suture region were analyzed using a t-test.
(TIF)

Figure S2 ***Gdf6*** **expression at E14.5.** Gdf6 expression was downregulated in the frontal bone by E14.5 in wild-type (B), *Gdf6+/−* (D), and *Gdf6−/−* embryos (F). Adjacent sections were stained for ALP to highlight the locations of the frontal and parietal bones (E–G, dotted lines). Previously reported *Gdf6* expression in the middle ear bone joints was clearly visible in sections from the same series, acting as a positive control (G,H).
(TIF)
