# Supplementary figures and images for: The BMP Ligand Gdf6 Prevents Differentiation of Coronal Suture Mesenchyme in Early Cranial Development
Source: PLoS One. 2012 May 31;7(5):e36789. doi: 10.1371/journal.pone.0036789 (PMC3365063; doi:10.1371/journal.pone.0036789)

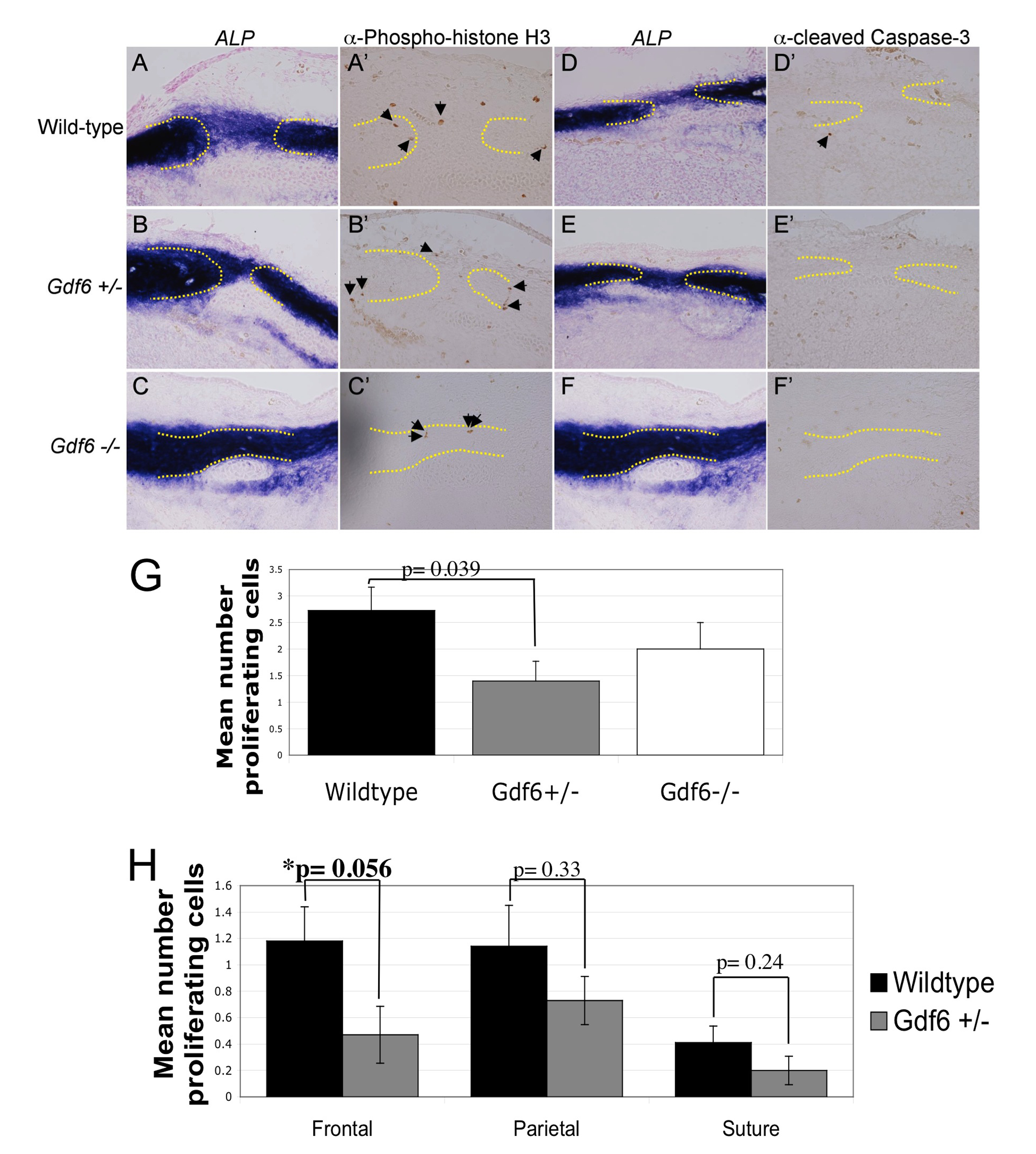

Supplement: Figure S1 — Analysis of cell proliferation and apoptosis in the coronal suture. Adjacent sections stained for ALP activity, highlighting the location of the frontal and parietal bones (A–F dotted lines), and antibodies for phospho-histone H3 (A’–C’) or cleaved caspase-3 (D’–F’). Positive cells are marked with arrows. (G) Quantification of the mean number of proliferating cells (y-axis) counted in the suture region (x-axis). (H) Quantification of the mean number of proliferating cells (y-axis) counted in each region of the suture; the frontal bone, parietal bone, and suture mesenchyme (x-axis). The mean number of proliferating cells in each region of the suture was not counted for Gdf6−/− embryos since there is no suture mesenchyme and the border between the frontal and parietal bones cannot be distinguished. N = 3 embryos for each genotype and antibody treatment with 5 sections per embryo quantified. Differences the number of proliferating cells per suture region were analyzed using a t-test. (TIF) [file pone.0036789.s001.tif]

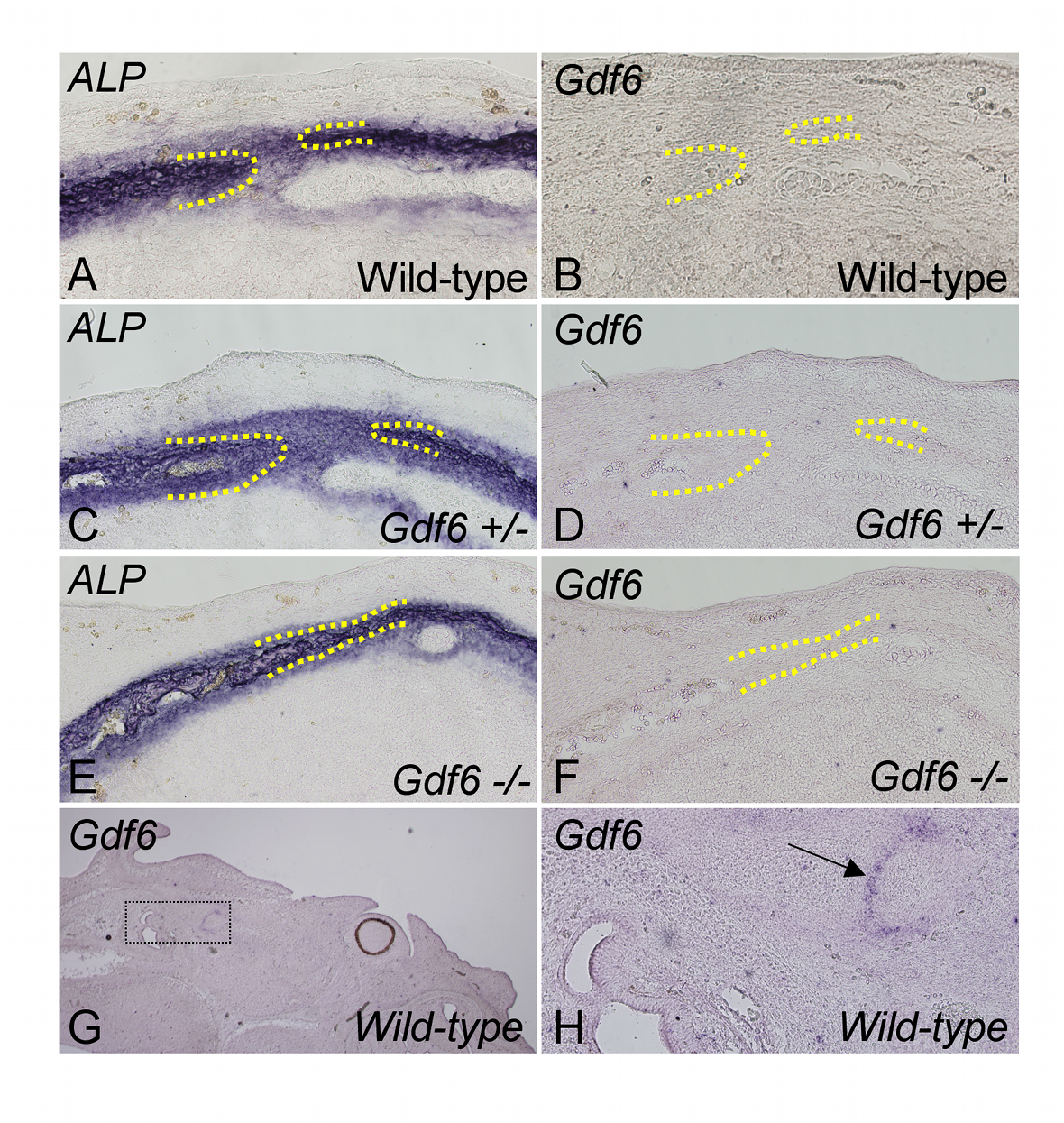

Supplement: Figure S2 — Gdf6 expression at E14.5. Gdf6 expression was downregulated in the frontal bone by E14.5 in wild-type (B), Gdf6+/− (D), and Gdf6−/− embryos (F). Adjacent sections were stained for ALP to highlight the locations of the frontal and parietal bones (E–G, dotted lines). Previously reported Gdf6 expression in the middle ear bone joints was clearly visible in sections from the same series, acting as a positive control (G,H). (TIF) [file pone.0036789.s002.tif]
